# Heart Rate Variability and Perceived Stress as Measurements of Relaxation Response

**DOI:** 10.3390/jcm8101704

**Published:** 2019-10-16

**Authors:** Mutsuhiro Nakao

**Affiliations:** Department of Psychosomatic Medicine, School of Medicine, International University of Health and Welfare, Narita, Chiba 286-8686, Japan; m-nakao@iuhw.ac.jp; Tel.: +81-476-20-7701

Stress is a term used to define the body’s physiological and psychological reactions to circumstances that require behavioral adjustment [1,2,3], and the relaxation response is a psychophysiological state that is opposite to that of the stress or fight–flight response [4]. A variety of mind/body techniques can be used to elicit a relaxation response and achieve the therapeutic effects associated with reduced blood pressure. For example, researchers at the Cochrane Review [5] investigated the following eight relaxation therapies for the management of primary hypertension in adults: autogenic training, behavioral therapy, biofeedback, breathing exercises, cognitive therapy, guided imagery, meditation, and progressive muscle relaxation. These approaches can be divided into three types of active intervention: biofeedback, cognitive/behavioral therapy (including guided imagery, meditation, and yoga), and autogenic training/progressive muscle relaxation. Although this Cochrane Review study concluded that the evidence for the causal association between relaxation and blood pressure reduction is weak [5], many previous studies [6,7,8] have reported that such relaxation techniques are useful for reducing sympathetic nervous activation and thereby decreasing blood pressure, heart rate, and other psychophysiological parameters in the service of reducing the harmful effects of stress. 

Heart rate variability, which refers to fluctuations in the length of the interval between heart beats, is commonly measured using the R–R interval on an electrocardiogram. It is one of the most sensitive and non-invasive indicators of autonomic nervous function. Power spectral analysis of sequential R–R intervals enables the classification of heart rate variability into three frequency bands: very low frequency (VLF < 0.04 Hz), low frequency (LF: 0.04–0.15 Hz), and high frequency (HF: 0.15–0.40 Hz). HF is usually considered to reflect parasympathetic nervous activity, and LF or LF/HF to reflect sympathetic nervous activity [6]. These parameters enable us to monitor autonomic nervous function successively during the relaxation response, and have been employed in stress-related studies.

The results of a meta-analysis of randomized controlled trials (RCTs) examining the effects of tai chi and yoga on heart-rate variability and perceived stress have recently been published. Tai chi, also called taiji or tai chi chuan, is a form of mind–body exercise originating in China. Yoga, which includes stretching, postural and breathing exercises, and meditation, originated in India. Both techniques have similar components such as the cultivation of mind/body connections through slow voluntary movements, diaphragmatic breathing practice, and meditative states of mental concentration [9]. According to the results of 17 RCTs using either of the two techniques, normalized HF was significantly increased (Hedge’s g: 0.39; 95% confidence interval: 0.22–0.52) in the intervention when compared with the control group, which means that the increased level of parasympathetic nervous function observed was attributable to the relaxation intervention. Additionally, LF/HF was significantly decreased (by −0.58; −0.81 to −0.35) by the intervention, which means that the decreased level of sympathetic nervous function can also be traced to the intervention. Moreover, the perceived stress level was significantly reduced (by −0.80; −1.17 to −0.44) by the intervention. The authors concluded that stress reduction may be attributed to the sympathetic–vagal balance modulated by the mind/body exercises associated with tai chi and yoga [9].

One of the main concerns raised by this article is whether it is appropriate to treat tai chi and yoga as a single technique. As the authors noted in the section discussing the limitations, yoga varies substantially across cultures. Although combining tai chi with yoga is consistent with the rough classification of the previous Cochrane Review study [5] and may be justified by the limited number of related extant RCTs, future research should involve RCTs in different settings across cultures. This would enable separate sub-analyses of tai chi and yoga to determine whether these techniques should be treated as identical in terms of the relaxation response. The present study focused on the use of these relaxation techniques to achieve psychological well-being, which is particularly important given that a recent meta-analysis [10] revealed that tai chi had significant beneficial effects on depression (effect size: −5.95; 95% confidence interval: 0.706–0.487). Indeed, one advantage of the present review [9] is its validation that perceived stress is significantly improved by tai chi and yoga.

Heart rate variability is closely related to mental status (e.g., degrees of anxiety and depression), hormonal response (e.g., cortisol and catecholamine plasma concentrations), cardiovascular system functions (e.g., blood pressure and heart rate), and lifestyle factors (alcohol consumption and smoking) [11]. It will become increasingly possible to monitor heart rate variability and biofeedback responses during the relaxation response as information and communication technologies advance.
